# Novel Approach for the Fabrication of Composite Rocket Propellant: Increased Homogeneity and Its Influence on SRP Behaviour

**DOI:** 10.3390/ma19050979

**Published:** 2026-03-03

**Authors:** Kinga Janowska, Marcin Procek, Tymon Warski, Mateusz Polis, Agnieszka Stolarczyk, Lukasz Hawelek

**Affiliations:** 1 Department of Physical Chemistry and Technology of Polymers, Silesian University of Technology, 44-100 Gliwice, Poland; kinga.janowska@polsl.pl; 2 Department of Optoelectronics, Silesian University of Technology, 44-100 Gliwice, Poland; marcin.procek@polsl.pl; 3 Institute of Non-Ferrous Metals, Lukasiewicz Research Network, 44-100 Gliwice, Poland; tymon.warski@imn.lukasiewicz.gov.pl (T.W.);; 4 Institute of Industrial Organic Chemistry, Lukasiewicz Research Network, 42-693 Krupski Młyn, Poland; 5 Institute of Aviation, Lukasiewicz Research Network, 02-256 Warszawa, Poland; mateusz.polis@ilot.lukasiewicz.gov.pl

**Keywords:** solid rocket propellant, electrospraying, microstructural homogeneity, porosity, thermal decomposition, energetic materials

## Abstract

In this study, the feasibility of electrospraying as an alternative processing technique for the preparation of composite solid rocket propellants (SRPs) was investigated. The main objective was to improve microstructural homogeneity and interfacial contact between the oxidizer, energetic additive, and metallic fuel without altering the chemical composition of the formulation. Additionally, porous electrosprayed SRP formulations were prepared to examine the influence of controlled porosity on thermal decomposition behavior. The prepared materials were characterized using scanning electron microscopy combined with energy-dispersive X-ray spectroscopy (SEM/EDS) to assess microstructural features and component distribution. Thermal decomposition behavior and kinetic parameters were evaluated using simultaneous DSC/TG analysis conducted at multiple heating rates. Safety-related properties were assessed through friction sensitivity testing, while post-decomposition solid residues were analyzed using SEM/EDS and X-ray diffraction. The results show that electrospraying improves structural homogeneity, reduces solid residue formation after thermal decomposition, and decreases apparent activation energy, while maintaining unchanged friction sensitivity. These findings demonstrate the potential of electrospraying as a physical processing route for tailoring the microstructure and thermal behavior of composite solid rocket propellants.

## 1. Introduction

Composite solid rocket propellants (SRPs) are a well-established class of energetic materials used in solid rocket motors, consisting of an oxidizer, a polymeric binder acting as both fuel and matrix, and optional energetic or metallic additives. The term “SRP” is used throughout this manuscript in its conventional meaning in solid rocket propulsion technology and does not refer to a newly defined or proprietary material. SRPs are a crucial class of energetic materials widely used in aerospace and defense applications due to their high energy density, reliability, and long-term storage capability [1,2,3,4]. Typical composite propellants consist of an oxidizer, most commonly ammonium perchlorate (AP), a polymeric binder such as hydroxyl-terminated polybutadiene (HTPB), and optional metallic or catalytic additives [5,6,7]. The performance and safety characteristics of these materials are strongly governed by their microstructure, including component distribution, interfacial contact, and porosity [2,8,9].

Porosity represents a critical microstructural feature in energetic composites [10,11]. Controlled pore formation may increase the internal surface area, facilitate gas diffusion during thermal decomposition, and modify reaction pathways observed in thermogravimetric analysis [5,12]. At the same time, excessive or poorly controlled porosity may negatively affect mechanical integrity and sensitivity of energetic formulations [13,14]. Consequently, processing routes that enable improved control over microstructure without chemical modification of energetic components remain of significant interest.

Traditional preparation methods for SRPs, such as mechanical hand mixing followed by casting and curing, often lead to heterogeneous microstructures and limited control over interfacial contact between components [1,2]. Numerous studies have focused on the use of nano-sized catalysts and additives to influence thermal decomposition of AP and AP/HTPB propellants [5,12,15,16,17]. Although such approaches are effective in modifying thermal behavior, they do not directly address microstructural homogeneity or pore architecture of the composite matrix.

Despite these advances, the application of electrospraying to composite solid rocket propellants remains limited. In particular, a systematic comparison betweenhand-mixed SRPs and electrosprayed structures, focusing on porosity formation, thermal decomposition kinetics, and combustion residue characteristics, has not yet been reported. The relationship between processing route, microstructural features observed by scanning electron microscopy, and kinetic parameters derived from thermogravimetric analysis remains insufficiently understood [18].

In the present work, electrospraying is investigated as an alternative processing route for the preparation of porous composite solid rocket propellants. Three different processing strategies, including hand-mixing and electrospraying-based approaches, are systematically compared. Microstructural features and component distribution are examined using scanning electron microscopy combined with energy-dispersive X-ray spectroscopy (SEM/EDS). Thermal decomposition behavior and activation energy values are evaluated using thermogravimetric analysis at different heating rates. In addition, the morphology and composition of combustion residues are analyzed to assess the influence of processing route on post-decomposition products. The obtained results provide insight into structure–property relationships relevant to the design of advanced energetic materials.

## 2. Materials and Methods

### 2.1. Materials

#### 2.1.1. Preparation of SRP Formulations

All experimental procedures involving energetic materials were conducted in dedicated laboratories authorized for handling explosive substances, in accordance with applicable fire and explosion safety regulations, and by trained personnel experienced in energetic materials research.

Manually mixed formulation (SRP-M) were prepared in a glass vessel by introducing individual components in a fixed order and mixing manually with a glass stirring rod until a macroscopically uniform paste was obtained. Because curing of the GAP binder system proceeded concurrently, preparation was conducted on a hot plate maintained at 60 °C. To limit moisture uptake by the oxidizer, the prepared SRP samples were stored in a drying oven at 50 °C prior to characterization. The compositions of the prepared SRP formulations are summarized in Table 1 and Table 2.

For electrosprayed formulation (SRP-E) and porous electrosprayed formulation (SRP-EP), the solid mixture of PSAN/NQ/Ti obtained by electrospraying was used as the energetic solid fraction. The solid phase obtained by electrospraying was collected as a dry powder and subsequently incorporated into the liquid binder by gradual addition under continuous mechanical stirring. This procedure ensured uniform dispersion of the electrosprayed solid components within the binder matrix prior to curing. For SRP-EP, low-boiling solvent after the synthesis of the binder was used as a foaming agent. The foaming process was carried out continuously at a temperature of 60 °C under constant stirring and stored in the drying oven.

#### 2.1.2. Electrospraying

**Figure 1:**
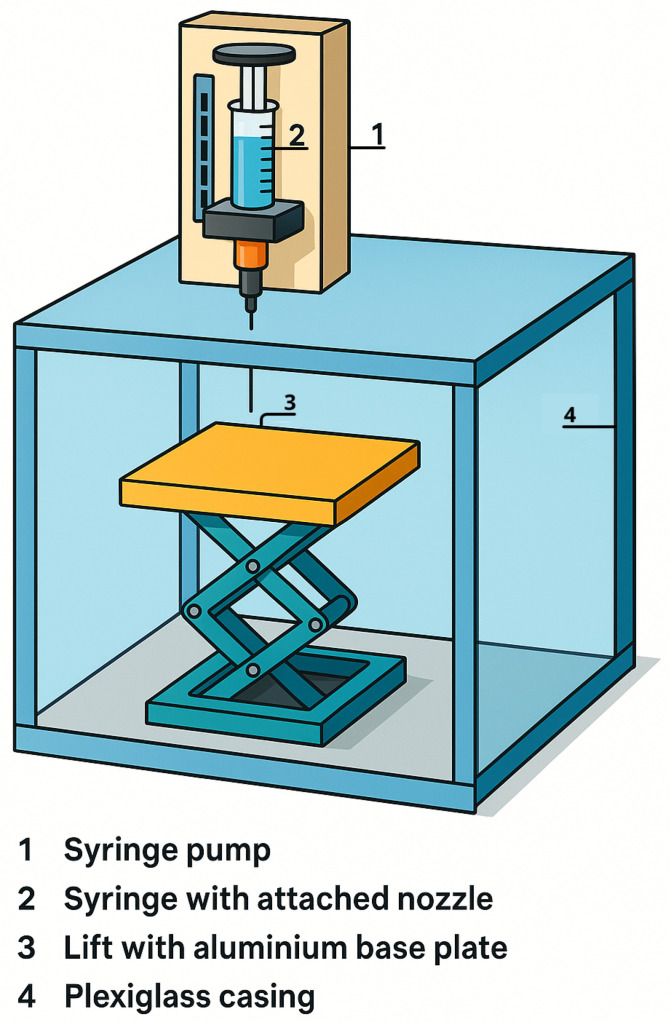
Schematic representation of the electrospraying apparatus.

The electrospraying setup consisted of an NE-1000 one-channel programmable syringe pump connected to a 25 kV high-voltage power supply (521721, Leybold, Cologne, Germany). A suspension containing the oxidizer (PSAN), energetic additive (NQ), and metallic fuel (Ti) was atomized under a high electric field (Figure 1) and collected on a grounded settling plate/collector, fabricated from aluminum. Methanol was used as the solvent. The solvent loading with solids was adjusted such that both PSAN and NQ dissolved, while Ti remained dispersed as a suspension in methanol. Partial dissolution was intentionally employed to reduce heterogeneity arising from the broad particle-size distribution of the raw materials.

The electrospraying process was carried out at an applied voltage of 19 kV at ambient conditions, as summarized in Table 3. The polarity of the applied voltage and the needle configuration were kept constant throughout the experiments (needle positive, collector grounded). Each electrospraying run was continued until the required amount of solid fraction was collected (each time 1.5 mL of the prepared suspension was sprayed).

### 2.2. Methods

#### 2.2.1. SEM/EDS Analysis

Morphology was investigated using a FEI Inspect S50 scanning electron microscope (FEI, Hillsboro, OR, USA) equipped with an Octane Elect Plus EDS detector (EDAX Inc., Mahwah, NJ, USA) and Apex Advanced software (Version 3.8.6.3964). Additional observations were conducted using a Phenom ProX SEM (Thermo Fisher Scientific, Waltham, MA, USA). Samples were placed on a conductive carbon tape and were sputter-coated with a 5 nm thick gold. Samples were examined under high vacuum $4.86\times 10^{-6}$ mbar, accelerating voltage was 5 kV and the working distance was 11 mm. EDS maps were recorded to evaluate relative elemental distribution.

#### 2.2.2. Determination of Friction Sensitivity

Friction sensitivity was determined using a Peters friction apparatus according to EN 13631-3 [14].

#### 2.2.3. Thermal Analysis and Kinetic Evaluation

Thermal behavior was investigated using simultaneous DSC/TG measurements performed with a Netzsch STA 449 F3 Jupiter thermal analyzer (Netzsch, Selb, Germany). Measurements were carried out from 20 °C to 1100 °C at heating rates of 3, 5, 7, 10, and 12 °C·min^−1^ in order to enable kinetic analysis using the Kissinger method. Individual TG/DTG curves obtained at different heating rates were used for activation energy calculations, while representative thermal profiles are shown for clarity. All measurements were performed under a controlled purge gas atmosphere (argon after a single evacuation of air, with a purge flow of 240.3 mL·min^−1^) using redAl_2_O_3_ crucibles (85 μL) with lids.

Apparent activation energy values reported in Table 5 were determined from the multi-rate TG/DSC data using the Kissinger method [21]. The kinetic analysis was employed for comparative evaluation of processing-route effects rather than for determination of absolute kinetic parameters.

#### 2.2.4. X-Ray Diffraction (XRD)

X-ray diffraction measurements were performed using Cu Kα radiation (λ=1.54183 Å) with a Rigaku MiniFlex 600 diffractometer (Rigaku Co., Tokyo, Japan) equipped with a one-dimensional detector (Rigaku D/teX Ultra 250) and a zero-background monocrystalline silicon sample holder. The X-ray tube was operated at 40 kV and 15 mA. Data were collected at room temperature over a 2θ range of 20°–80° using an IHS slit of 2 mm, Soller slits of 2.5°, a DS slit of 1.25°, a step size of 0.01°, and a counting time of 1.67 s per step, without sample rotation. Phase identification was conducted using PDXL2 Version 2.9.2.0 software and reference patterns from the ICDD PDF-2 database (Release 2025).

## 3. Results

### 3.1. Microstructural Characterization (SEM/EDS)

**Figure 2:**
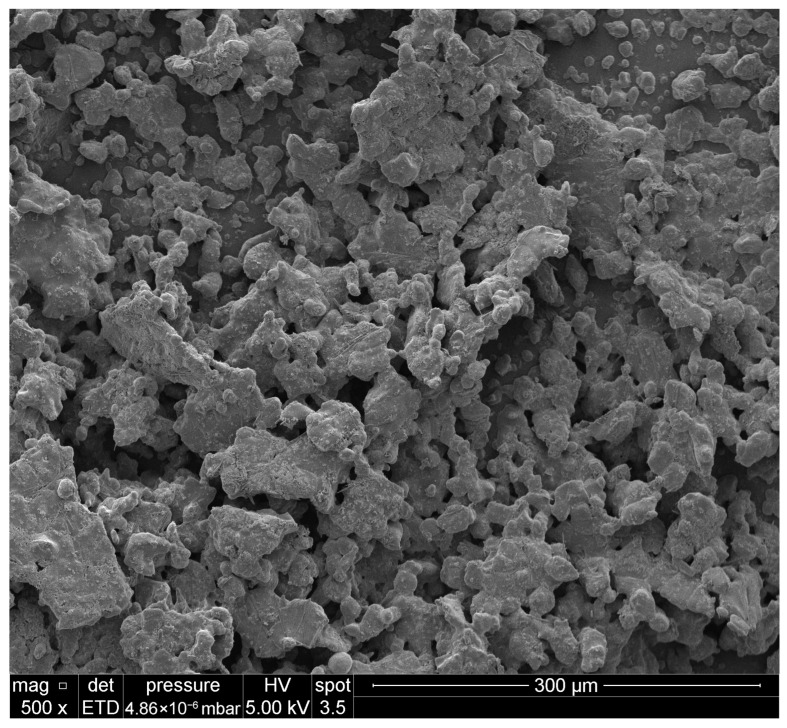
Structures obtained by electrospraying.

To evaluate the influence of the processing route on the microstructure of the investigated solid rocket propellants, scanning electron microscopy (SEM) analysis was performed. Representative SEM images of the structures obtained by electrospraying are shown in Figure 2. The electrosprayed samples exhibit a microstructure characterized by increased interfacial contact between individual components and the presence of distributed porosity, in contrast to the more compact morphology observed for hand-mixed materials.

SEM images reveal that the electrospraying process leads to the formation of structures with an increased contact surface between individual components (Figure 2). Difficulties in achieving adequate homogeneity in hand-mixed SRP formulations are primarily associated with the presence of nitroguanidine (NQ) in the form of elongated needle-like crystals, which hinder uniform dispersion within the polymer matrix. The application of electrospraying enabled the formation of significantly smaller NQ crystallites, likely with altered morphology, resulting in improved dispersion and enhanced compositional uniformity of the propellant formulations.

**Figure 3:**
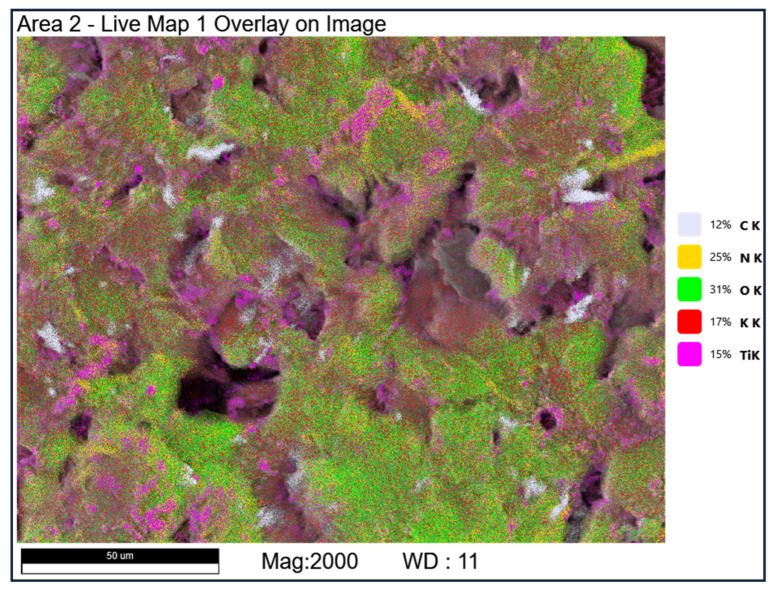
SEM/EDS mapping of electrosprayed mixture.

**Figure 4:**
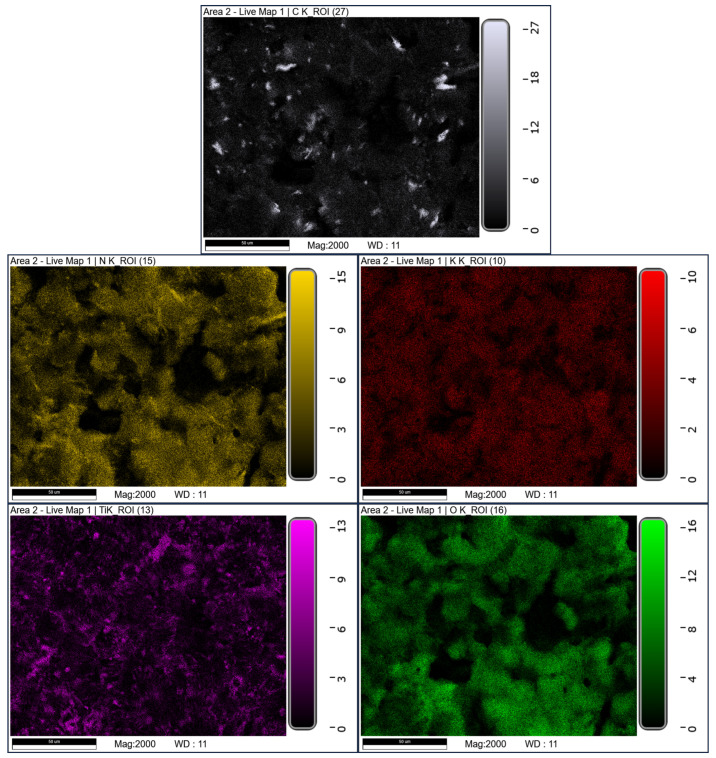
SEM/EDS-based elemental mapping of electrosprayed mixture.

SEM/EDS elemental mapping confirms an increased interfacial contact between PSAN, nitroguanidine, and titanium in the electrosprayed formulations (Figure 3 and Figure 4). In this study, increased interfacial contact refers to a qualitative microstructural observation based on SEM and SEM/EDS analyses, manifested by reduced phase segregation, finer dispersion of energetic components, and the absence of distinct interfacial gaps between the oxidizer, energetic additive, and metallic fuel. No quantitative interfacial area measurements were attempted. Elemental distribution maps of C, N, K, O, and Ti indicate a relatively uniform spatial distribution of all components. No pronounced local agglomeration or element-enriched regions were observed, suggesting a high degree of sample homogenization. Due to the semi-quantitative nature of EDS analysis, these results are interpreted in terms of relative elemental distribution rather than absolute composition.

**Figure 5:**
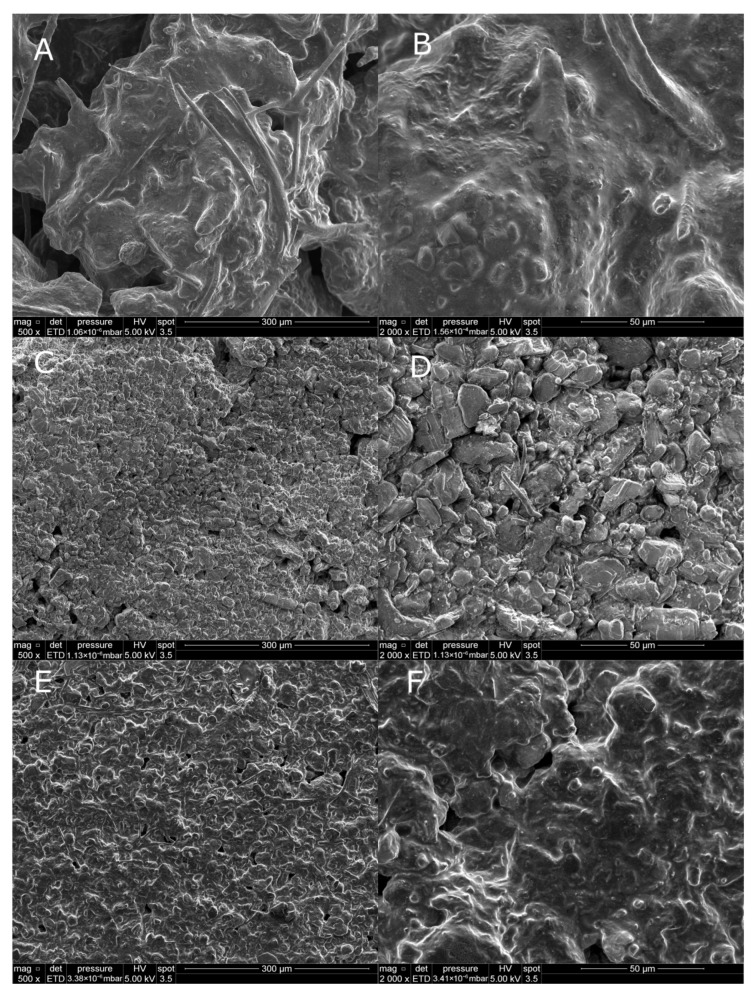
Morphology of tested SRP formulations ((**A**) 500× magnification of SRP-M; (**B**) 2000× magnification of SRP-M; (**C**) 500× magnification of SRP-E; (**D**) 2000× magnification of SRP-E; (**E**) 500× magnification of SRP-EP; (**F**) 2000× magnification of SRP-EP).

Further SEM analysis demonstrates that the processing route has a decisive influence on the morphology of the investigated SRP formulations. The manually mixed sample (SRP-M) exhibits a heterogeneous structure with irregular agglomerates unevenly distributed within the matrix (Figure 5A,B). Localized clusters of nitroguanidine are visible, indicating limited interfacial contact between individual components. Such morphology is characteristic of hand mixing and may restrict effective heat and mass transfer during thermal decomposition. Local agglomeration of energetic components and limited interfacial contact can lead to non-uniform heat distribution, delayed diffusion of gaseous decomposition products, and the formation of locally isolated reaction zones. As a consequence, thermal decomposition may proceed in a less homogeneous manner, promoting incomplete reactions and increased solid residue formation. Similar effects of microstructural heterogeneity on thermal and reactive behavior have been widely reported for mechanically mixed and foamed composite solid propellants [8,9]

In contrast, the electrosprayed formulation (SRP-E) exhibits significantly improved structural homogeneity (Figure 5C,D). The components are more uniformly distributed, and no distinct regions enriched in individual components are observed. This suggests that electrospraying effectively reduces phase segregation and increases interfacial contact between the oxidizer, energetic additive, and metallic fuel.

The most favorable morphology is observed for the porous electrosprayed formulation (SRP-EP) (Figure 5E,F). In addition to uniform component distribution, a system of micro- and mesopores is observed across the sample surface. This porous architecture increases the specific surface area and improves interfacial contact, which may facilitate heat and mass transfer during thermal decomposition. In the case of the porous electrosprayed formulation (SRP-EP), the statement regarding increased surface area is based on qualitative microstructural observations from SEM analysis, specifically the presence of micro- and mesoporous features and a more open architecture compared to non-porous samples. No quantitative determination of specific surface area (e.g., BET analysis) was performed. Therefore, the term “increased surface area” is used here to indicate a relative increase in accessible surface associated with porosity rather than an absolute specific surface area value.

It should be noted that the SEM analysis presented in this study is primarily qualitative and intended to compare relative microstructural features between different processing routes rather than to provide absolute porosity values.

### 3.2. Friction Sensitivity Testing

Friction sensitivity tests revealed identical values for the hand-mixed (SRP-M) and electrosprayed (SRP-E) formulations, both equal to 120 N (Table 4). This indicates that the change in preparation method, despite improving component distribution and microstructural uniformity, did not lead to an increase in friction sensitivity of the investigated SRP systems.

In contrast, the porous electrosprayed formulation (SRP-EP) exhibited a lower friction sensitivity (80 N). This behaviour can be attributed to the presence of a porous structure, which may promote local energy concentration and enhanced frictional interaction under used load.

These results suggest that friction sensitivity is more strongly influenced by the intrinsic properties and local distribution of solid energetic components, such as titanium and nitroguanidine, than by the overall increase in interfacial contact area. Moreover, friction sensitivity is governed not only by the overall degree of microstructural homogeneity but also by the mechanical characteristics of the material, including porosity.

### 3.3. Thermogravimetric Analysis

**Figure 6:**
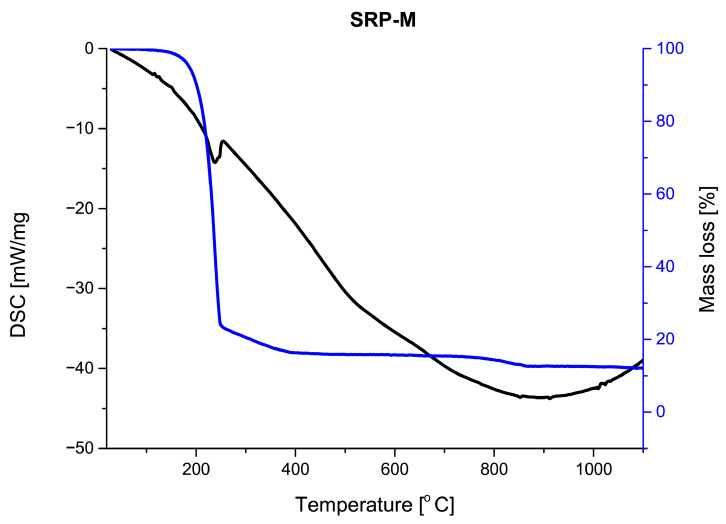
DSC/TG analysis of SRP-M.

**Figure 7:**
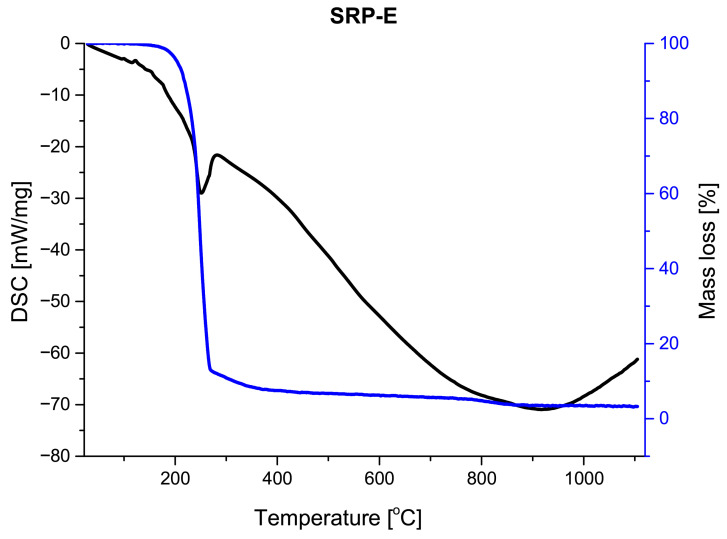
DSC/TG analysis of SRP-E.

**Figure 8:**
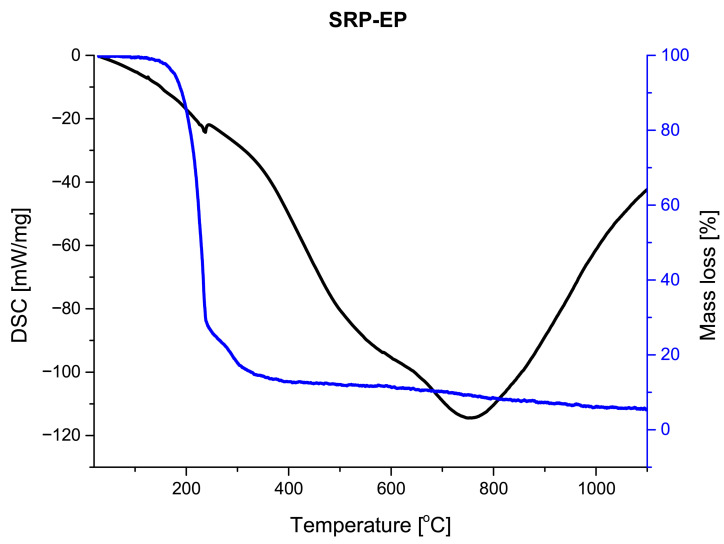
DSC/TG analysis of SRP-EP.

Thermogravimetric (TG) and derivative thermogravimetric (DTG) profiles obtained for the investigated SRP formulations (Figure 6, Figure 7 and Figure 8) show that all samples undergo their main mass loss within a similar temperature range. This observation suggests that the overall decomposition mechanism of the formulations remains unchanged regardless of the processing route.

The most pronounced differences between the investigated formulations are observed in the amount of solid residue remaining after thermal decomposition. The hand-mixed formulation (SRP-M) exhibits the highest residue fraction, exceeding 10%, which may be attributed to its heterogeneous microstructure and limited interfacial contact. In contrast, the electrosprayed formulations (SRP-E and SRP-EP) show significantly lower residue fractions, not exceeding 5%. In the case of SRP-EP, the presence of a porous structure further promotes efficient thermal decomposition by facilitating gas diffusion. The SRP-EP exhibits a distinct influence on the thermal behavior of the investigated composites. The introduction of porosity primarily affects the efficiency and homogeneity of the thermal decomposition process by facilitating heat and mass transfer, which is reflected in the reduced amount of solid residue after decomposition. The thermal stability of the formulations was assessed based on the temperature corresponding to the maximum mass loss rate (DTG peak temperature), which is commonly used as an indicator of the intrinsic thermal stability of composite propellants. The DTG peak temperature for the hand-mixed formulation (SRP-M) occurs at approximately $T_{\mathrm{DTG},\mathrm{SRP}\text{-}M}\approx 241\;{}^{\circ}C$, while for the electrosprayed formulation (SRP-E) it is observed at $T_{\mathrm{DTG},\mathrm{SRP}\text{-}E}\approx 235\;{}^{\circ}C$. In the case of the porous electrosprayed formulation (SRP-EP), the DTG peak is located at $T_{\mathrm{DTG},\mathrm{SRP}\text{-}\mathrm{EP}}\approx 234\;{}^{\circ}C$.

The comparable DTG peak temperatures indicate that the introduction of porosity does not adversely affect the intrinsic thermal stability of the composite. Instead, the role of SRP-EP is mainly associated with modifying the decomposition kinetics and improving the efficiency of the thermal degradation process, rather than shifting the main decomposition temperature.

### 3.4. Determination of Activation Energy

The apparent activation energy ($E_{a}$) values determined for the investigated formulations (Table 5) indicate a clear dependence of thermal decomposition behavior on the processing route. Compared to the hand-mixed reference (SRP-M, 137 kJ/mol), a reduced activation energy is observed for the electrosprayed formulation (SRP-E, 130 kJ/mol). This reduction is attributed to improved microstructural homogeneity and increased interfacial contact between energetic components.

The lowest activation energy is observed for the porous electrosprayed formulation (SRP-EP, 120 kJ/mol), indicating that the introduction of porosity further facilitates the initiation of decomposition reactions. The kinetic analysis is intended to provide a comparative assessment of the influence of processing route on decomposition behavior rather than to determine absolute kinetic parameters.

### 3.5. Post-Decomposition Residue Analysis (SEM/EDS and XRD)

**Figure 9:**
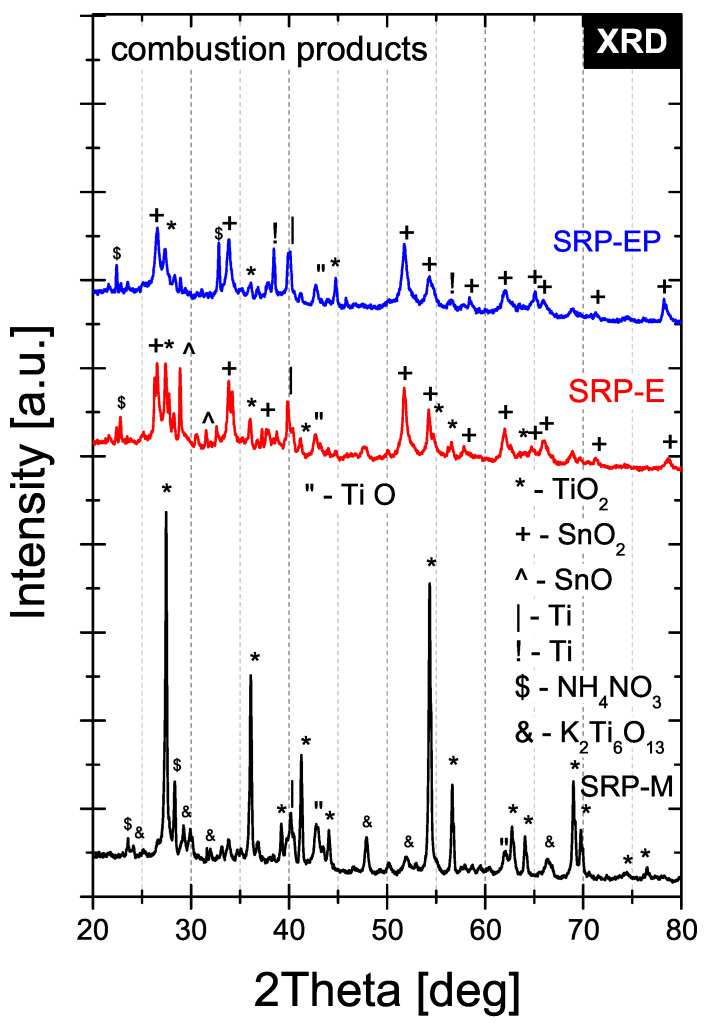
XRD patterns of solid residues after thermal decomposition.

XRD patterns of the solid residues obtained after thermal decomposition are shown in Figure 9. For SRP-M, rutile TiO_2_ is the dominant phase, accompanied by K_2_Ti_6_O_13_, metallic Ti, TiO, and residual NH_4_NO_3_. In contrast, residues from the electrosprayed formulations (SRP-E and SRP-EP) consist primarily of rutile TiO_2_, metallic Ti, and SnO_2_, with minor contributions from NH_4_NO_3_ and TiO.

The presence of SnO_2_ in all samples is attributed to the decomposition of the curing catalyst and does not indicate incomplete decomposition of the propellant formulation.

**Figure 10:**
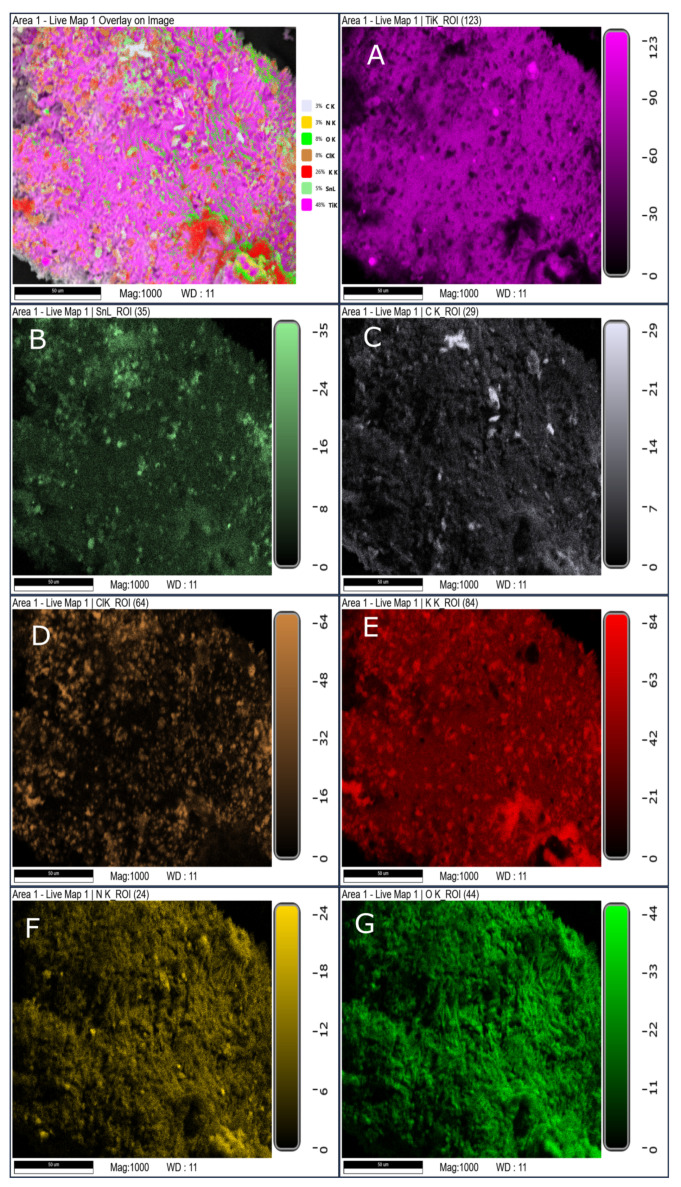
SEM/EDS mapping of solid residue of SRP-M (where (**A**) Element map of Ti; (**B**) element map of Sn; (**C**) Element map of C; (**D**) Element map of Cl; (**E**) Element map of K; (**F**) Element map of N and (**G**) Element map of O).

**Figure 11:**
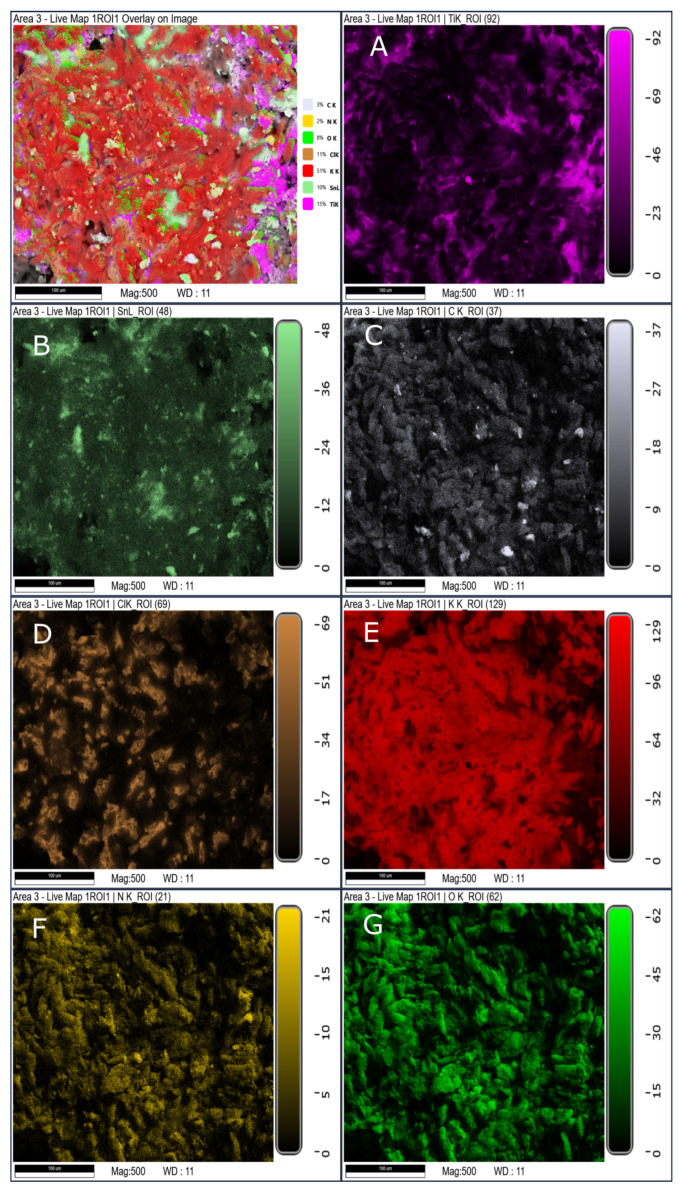
SEM/EDS mapping of solid residue of SRP-E (where (**A**) Element map of Ti; (**B**) element map of Sn; (**C**) Element map of C; (**D**) Element map of Cl; (**E**) Element map of K; (**F**) Element map of N and (**G**) Element map of O).

**Figure 12:**
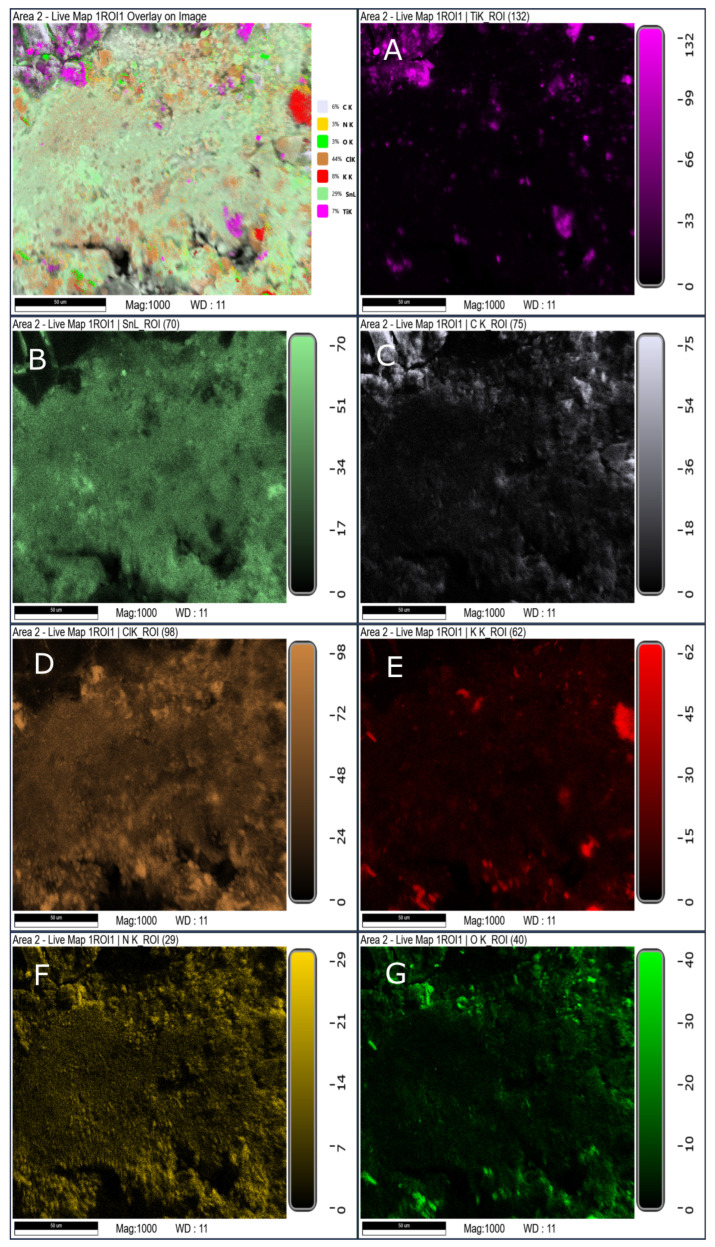
SEM/EDS mapping of solid residue of SRP-EP (where (**A**) Element map of Ti; (**B**) element map of Sn; (**C**) Element map of C; (**D**) Element map of Cl; (**E**) Element map of K; (**F**) Element map of N and (**G**) Element map of O).

SEM/EDS mapping of the solid residues further confirms that the processing route significantly influences the spatial distribution of reaction products (Figure 10, Figure 11 and Figure 12). The hand-mixed formulation (SRP-M) exhibits heterogeneous residue morphology with localized regions enriched in titanium- and oxygen-containing phases, consistent with the complex phase composition identified by XRD.

In contrast, residues from the electrosprayed formulation (SRP-E) display a more uniform elemental distribution, indicating improved homogeneity of the decomposition process. The porous electrosprayed formulation (SRP-EP) exhibits the most homogeneous residue morphology, with no distinct localized enrichment zones observed. This suggests that porosity facilitates more uniform heat and mass transfer during thermal decomposition. From a processing perspective, electrospraying is inherently compatible with continuous and scalable electrohydrodynamic techniques, suggesting potential applicability beyond laboratory scale, provided that appropriate process control and safety measures are implemented.

## 4. Conclusions

In this work, electrospraying was investigated as an alternative processing route for the preparation of composite solid rocket propellants with tailored microstructure. Based on the obtained experimental results, the following conclusions can be drawn:

- Electrospraying enables effective modification of SRP microstructure, leading to improved homogeneity and increased interfacial contact between the oxidizer, energetic additive, and metallic fuel compared to hand-mixed formulation, as confirmed by SEM and SEM/EDS analyses.
- The electrosprayed formulations exhibit reduced segregation of individual components, while the introduction of porosity further enhances the uniformity of the internal architecture by increasing the specific surface area and facilitating heat and mass transfer during thermal decomposition.
- Friction sensitivity measurements demonstrate that the application of electrospraying does not adversely affect the safety characteristics of the investigated SRP formulations, with friction sensitivity values remaining unchanged relative to the hand-mixed reference material.
- Thermogravimetric analysis indicates that the overall decomposition mechanism of the tested formulations remains unchanged; however, electrospraying and porosity generation significantly reduce the amount of solid residues formed after thermal decomposition.
- Kinetic analysis based on multi-rate thermogravimetric data reveals a systematic decrease in apparent activation energy for electrosprayed and porous electrosprayed formulations, highlighting the influence of microstructural modification on the energetic decomposition process.
- Post-decomposition residue analysis using SEM/EDS and XRD confirms that electrospraying combined with porosity generation leads to a more homogeneous distribution of solid reaction products and a simplified phase composition compared to the hand-mixed formulation.

Overall, the results demonstrate that electrospraying constitutes a viable physical processing strategy for controlling the microstructure and thermal behavior of composite solid rocket propellants without altering their chemical composition. This approach provides a complementary pathway to hand-mixed preparation of SRPs formulation techniques and offers potential for future studies focused on structure–property relationships and advanced energetic material design.

## Figures and Tables

**Table 1:** Materials used in this work.

Chemical (Code)	Purity Grade	Source	Notes
Phase-stabilised ammonium nitrate (PSAN)	–	In-house preparation	Prepared as described in [19].
Glycidyl azide polymer (GAP)	–	In-house synthesis	Synthesised in-house; procedure based on polymer designs described in [20].
Nitroguanidine (NQ)	–	In-house synthesis	Synthesised in-house following a literature-guided procedure [13].
Hexamethylene diisocyanate (HMDI)	>95%	Sigma-Aldrich (Burlington, MA, USA)	Cross-linking agent.
Titanium (Ti)	99.9%	Iolitec GmbH (Heilbronn, Germany)	Nominal particle size: 50 nm.
Dibutyltin dilaurate (DBTDL)	>95%	Sigma-Aldrich (Burlington, MA, USA)	Catalyst for curing reaction.

**Table 2:** Constituents of tested rocket propellant formulations.

Components	Content (wt%)
PSAN	73
Glycidyl azide polymer (GAP)	13
Titanium (Ti)	10
Nitroguanidine (NQ)	2
Hexamethylene diisocyanate (HMDI)	2

**Table 3:** Electrospraying parameters.

Parameter	Value	Unit
Flow rate	3	cm^3^/h
Applied voltage	19	kV
Nozzle–collector distance	10	cm
Nozzle inner diameter	$4.3\times 10^{-2}$	cm
Solids loading	47	mg/mL

**Table 4:** Determined friction sensitivity.

Sample	Determined FS [N]
SRP-M ^1^	120
SRP-E ^2^	120
SRP-EP ^3^	80

^1^ SRP formulation obtained by mechanical mixing. ^2^ SRP formulation obtained using electrosprayed mixture. ^3^ Porous SRP formulation obtained using electrosprayed mixture.

**Table 5:** Determined activation energy values.

Sample	Determined $E_{a}$ [kJ/mol]
SRP-M ^1^	137
SRP-E ^2^	130
SRP-EP ^3^	120

^1^ SRP formulation obtained by mechanical mixing. ^2^ SRP formulation obtained using electrosprayed mixture. ^3^ Porous SRP formulation obtained using electrosprayed mixture.

## Data Availability

The original contributions presented in this study are included in the article. Further inquiries can be directed to the corresponding author.
